# BioGlue and Peri-strips in lung volume reduction surgery: pilot randomised controlled trial

**DOI:** 10.1186/1749-8090-4-37

**Published:** 2009-07-17

**Authors:** Sridhar Rathinam, Babu V Naidu, Prakash Nanjaiah, Mahmoud Loubani, Maninder S Kalkat, Pala B Rajesh

**Affiliations:** 1 Regional Department of Thoracic Surgery, Birmingham Heartlands Hospital, Bordesley Green East, Birmingham, UK

## Abstract

**Background:**

Both tissue sealants and buttressing have been advocated to reduce alveolar air leaks from staple lines following Lung Volume Reduction Surgery (LVRS). However, the long term detrimental effects of buttressing material are increasingly apparent. We performed a pilot prospective randomised self controlled trial in patients undergoing LVRS comparing BioGlue and Peri-strips as adjuncts in preventing alveolar air-leaks.

**Methods:**

A pilot prospective self controlled clinical trial was conducted in patients undergoing LVRS. Each patient was treated with BioGlue on one side and pericardial buttress on the other side as an adjunct to the staple line. The sides were randomised for adjuncts with each patient acting as his own control. Duration of air leak, intercostal drainage and time to chest drain removal were the study end points.

**Results:**

10 patients undergoing the procedure were recruited between December 2005 and October 2007. There were 6 men and the mean age was 59.8 ± 4.9 years. There was one mortality due to multi-organ failure. The BioGlue treated side had a shorter mean duration of air-leak (3.0 ± 4.6 versus 6.5 ± 6.9 days), lesser chest drainage volume (733 ± 404 ml versus 1001 ± 861) and shorter time to chest drain removal (9.7 ± 10.6 versus 11.5 ± 11.1 days) compared with Peri-strips.

**Conclusion:**

This study demonstrates comparable efficacy of BioGlue and Peri-strips, however there is a trend favouring the BioGlue treated side in terms of reduction in air-leak, chest drainage volumes, duration of chest drainage and significant absence of complications. A larger sample size is needed to validate this result.

## Introduction

Lung Volume Reduction Surgery (LVRS) benefits a selected group of patients with end stage emphysema [1,2]. A common and troublesome complication of LVRS is postoperative air leak aggravated by the friable nature of the underlying lung parenchyma [3,4]. A number of techniques have been utilised to prevent and minimise air leak, including buttressing materials such as bovine pericardium (Peri-strips) (Synovis Life Technologies Inc, St Paul MN, USA), poly-tetrafluoroethylene (PTFE), Teflon, polyglycollic acid and gel foam [3,5,6]. In addition, a number of surgical sealants are in use to achieve pneumostasis after pulmonary surgery [3,7-10].

BioGlue surgical sealant (CryoLife Inc. Kennesaw, U.S.A) is a topically applied mixture of bovine serum albumin and glutaraldehyde. It is approved for use as an adjunct to standard methods of haemostasis and for use in a wide range of soft tissue repairs. BioGlue has also been shown to reduce air leaks, length of chest drains and hospital stay in thoracic surgical practice [9]. However, at present there are no published reports regarding the utility or efficacy of BioGlue in LVRS patients.

The principal aim of our pilot randomised self controlled trial was to compare the use of BioGlue and buttressed pericardial strips in controlling postoperative air leak following LVRS. This was assessed by clinically relevant outcome measures of duration of air leak, drainage volume and time to drain removal. We report the results of our pilot phase in this paper.

## Materials and methods

### Patient Selection

A prospective, randomised self controlled trial with the approval of the East Birmingham Research Ethics Committee was performed at Birmingham Heartlands Hospital between December 2005 and October 2007. We used the CONSORT checklist for design and conduct of this study. As there are no comparative trials comparing these interventions we designed the study in two phases a pilot phase of ten patients to review the results and to calculate a sample size for the trial. An informed patient consent was obtained before each operation. Patients undergoing bilateral LVRS through a median sternotomy incision were included in the study. Patients with asymmetrical disease and known allergies to bovine pericardium and albumin were excluded. All patients had routine work-up for LVRS according to our unit protocol, comprising full lung function tests, high resolution CT scanning (HRCT), quantitative ventilation-perfusion scanning, echocardiography in patients with previous cardiac history, smoking cessation and pulmonary rehabilitation.

### Randomisation

**Figure 1:**
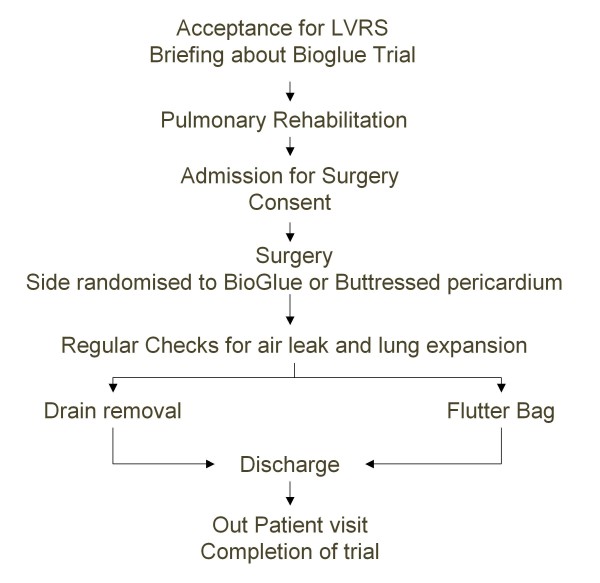
**Study Protocol**.

Randomisation was undertaken with sequential closed envelopes containing the treatment strategies assigned to each side. In each case, the operating surgeon opened the envelope on the day of surgery to assign the treatment strategy to each side (Figure 1).

### Surgery

All patients had a peri-operative epidural catheter sited for pain relief. The surgery was performed under general anaesthesia with a double lumen endotracheal tube and sequential single lung ventilation. The surgical access was gained through median sternotomy. The target areas were identified based on preoperative CT scan, ventilation perfusion scan as well as by direct per-operative observation and palpation of lung parenchyma. The pleural cavity was entered after instituting single lung ventilation to the contra lateral side. After few minutes of suspending ventilation, the relatively better part of the lung parenchyma tends to collapse thus demarcating the worst areas for excision. The line of excision began at the medial aspect, near the horizontal fissure for the right upper lobe and near the base of the lingula for the left upper lobe. With successive applications of the GIA 80 stapler (Auto Suture, Tyco Healthcare Norwalk CN, USA), the line of excision was carried up toward the apex, angled postero-laterally, and then angled downward on the postero-lateral portion of the upper lobe ending up near the top of the oblique fissure. Thus an inverted 'U' shaped continuous staple line was formed. The resection was limited to the level of the azygos vein on the right and to the aortic arch on the left.

### Intervention

BioGlue was applied on the stapled margins using a spreader tip. The unique double-helix delivery system attached to a syringe enables swift mixing of two components of the product. The lung was gently re-inflated after two minutes. In the other group Peri-strips were applied onto the jaws of the GIA stapler before applying them on the lung parenchyma.

### Checking for air leak

After ensuring pneumostasis, 28F apical and basal drains were placed in the pleural space and brought out through separate stab incisions. The pleural openings on either side were closed with a continuous run of 4/0 prolene suture (Ethicon USA) to prevent crossover air leak. Once the pleurae were isolated the air-leak check was repeated to rule out crossover air leaks.

The anaesthetist carefully avoided over-inflation of the operated lung letting the lung inflate gently accepting a period of relative hypercarbia. At the end of the procedure, the sternum was closed with sternal wires and the pre-sternal fascia and skin were closed in layers. The patients were all extubated at the end of the operation and nursed in the thoracic high dependency unit.

### Postoperative Care

The chest drains were connected to underwater seal drainage systems individually and left without suction. The drains were connected to flutter valve bags (Portex Ltd, Hythe UK) once the drainage was less than 100 mls/day to enable mobilisation. The chest drains were removed following cessation of the air leak, confirmation of full expansion of the lung and drainage less than 100 ml in 24 hours. This was achieved in majority of patients. However, if there was persistent air leak or the lung failed to expand after seven days, the patient was discharged home with drain connected to a flutter valve bag, which was removed subsequently. The patients were routinely followed up to 6 weeks after surgery.

In this study, the postoperative air leak was defined as the presence of air bubbles in the chest drains during the course of normal or forced expiration (coughing). The investigators performed daily objective assessments that were concurrently verified by independent blinded senior nursing staff.

### Statistical Analysis

The results are expressed as mean ± standard deviation. The sides randomised to each arm were grouped according to treatment allocation, compared with t tests for normally distributed measures and Mann-Whitney tests for non-normally distributed measures.

## Results

### Demographics

A total of 10 patients were recruited and consented over a period of 24 months into this study. There were 6 men with a mean age of 59.8 ± 4.9 years. All patients had discontinued smoking prior to surgery. The median Karnofsky performance scale of this group was 70%. Co-morbidities included hypertension (n: 1), diabetes mellitus (n: 1) and aortic regurgitation (n: 1). There was no significant difference in the distribution of emphysema between the two treatment sides as assessed by independent review of HRCT and quantitative ventilation perfusion imaging. A summary of pre-operative investigations is presented in Table 1.

**Table 1:** Demographics and Investigations

**Variable**		
**Age (Mean ± SD)**		59.8 ± 4.9 years
**Sex (M:F)**		6:4
**Predicted FEV1**	**% Mean (Range)**	22 (19–47)
**Residual Volume**	**% Mean (Range)**	225 (172–260)
**KCO**	**% Mean (Range)**	36 (15–58)
**Lung Volume Resected in cm^3 ^Mean (Range) Bioglue:Peri-strips**		194 (47–504):174 (46–365)

### Cessation of Air-leak

**Figure 2:**
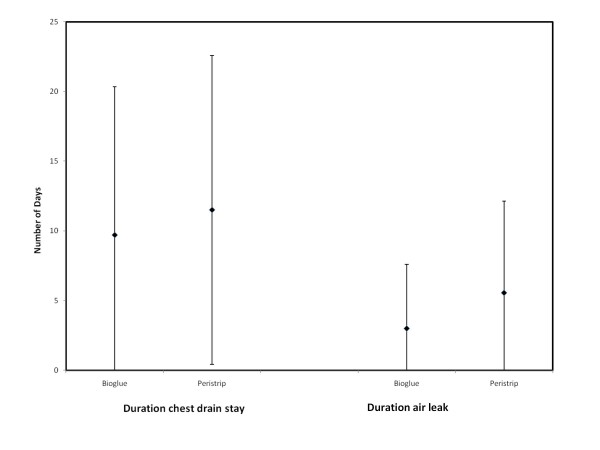
**Comparison of duration of air leak and chest drainage in the two groups**. (mean ± standard deviation).

**Figure 3:**
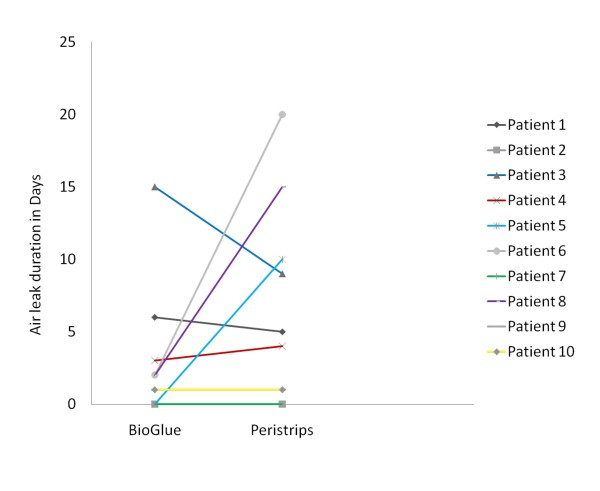
**Comparative results of BioGlue and Peri-strips in each patient**. The graph represents the number of days with air leak in the BioGlue arm on the left side and Peri-strips arm on the right side and links each patient with a bar. Two patients with identical values are superimposed on one another.

There was shorter mean duration of air leak, less drainage and chest drains were removed earlier in the BioGlue group but these do not reach statistical significance due to small numbers (Figure 2, Table 2). There was a tendency for earlier cessation or comparable duration of air leak in the BioGlue arm on direct comparison between the two treatment sides in all but one patient. Two patients did not have any post operative air leak. BioGlue treated sides had comparable duration of air leak with Peri-strips treated sides. In three patients the BioGlue treated side had significantly shorter air-leak duration and one patient had a longer air-leak in the BioGlue treated side (Figure 3).

**Table 2:** Comparative outcome between the groups

	BioGlue	Peri-strips	P
Air leak (days)	3.0 ± 4.6	6.50 ± 6.88	0.27
Drainage Volume(ml)	733 ± 404	1001 ± 861	0.65
ICD duration (days)	9.7 ± 10.6	11.5 ± 11.1	0.73

**Figure 4:**
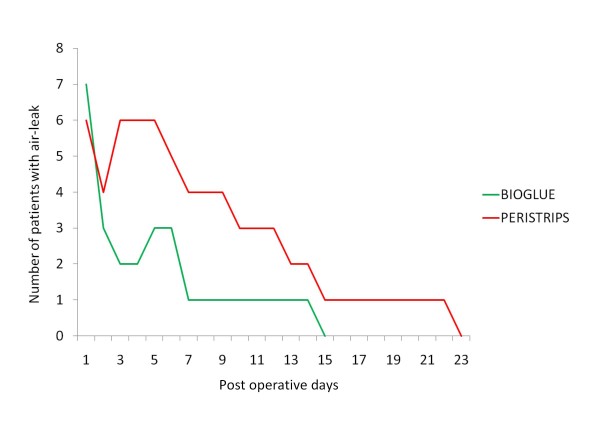
**Early cessation of air leak in the BioGlue arm on a day to day basis**.

When comparing the number of subjects with air-leak on each of the post operative days there was only one patient in the BioGlue treated side who had prolonged air leak (duration more than 7 days) compared with four in the Peri-strips treated side (Figure 4).

### Complications

There was single mortality on the 38^th ^post operative day, due to postoperative respiratory failure leading to multi-organ failure.

No adjunct related complications were encountered in the BioGlue arm. In Peri-strips side, one patient had air leak at the end of the procedure from the junction of two staple lines and required extra pneumostasis with insertion of prolene sutures. Although this can be viewed as technical failure, it may still be considered as adjunct failure. BioGlue application on the staple confluence may have sealed the leak where as with the Peri-strips it is incorporated into the overlapped stapling lines therefore necessacitating extra sutures. One patient had prolonged air leak on the Peri-strips treated side and was discharged home on a flutter valve bag. Another patient coughed up part of the Peri-strips three months after the surgical procedure.

## Discussion

Lung Volume Reduction Surgery for emphysema has evolved over the last two decades since the original description by Brantigan [11]. Cooper and colleagues popularised the use of stapled excision of the emphysematous lung with good outcomes [1]. This was followed by a number of groups pursuing varied selection criteria and techniques with mixed results [4,12-14]. However, the selection criteria and benefits of LVRS in end stage emphysema has been established in the National Emphysema Treatment Trial [2] with durable long term results in select group of patients [15].

One of the major complications of stapled LVRS is prolonged air leak which occurs in 50–90% of the patients [3]. A number of adjuncts to prevent air leak have been advocated which include bovine pericardium, Gore-Tex or autologous pleura [3,6]. The buttressing of the staple line has been shown to reduce the duration of air leak and time to chest drain removal [6]. In our centre, the standard approach to Lung Volume Reduction Surgery is through a median sternotomy incision and bilateral stapled excision with Peri-strips buttressing. We refrained from performing thoracoscopic LVRS because of the lack of endoscopic buttressing materials at the time of designing this study.

Though the buttressing adjuncts result in better pneumostasis, there are many documented cases of migration of the buttressing pericardium [5,16,17] or associated staples [18,19] sometimes resulting in harm to the patient. Following LVRS, we believe that the staple line causes a shearing force on the lung parenchyma which results in damage to the lung contributing to the air leak. Alternative adjuncts used for pneumostasis are tissue sealants and glues which are applied on the staple line and adjacent normal lung to prevent the shearing tears on the lung [20]. Although BioGlue has been used in certain centres in the NETT trial it was used as an additional adjunct to buttressed staple line [3]. However its role as a sole pneumostatic agent has not been tested in the LVRS setting. This randomised controlled trial compared BioGlue and Peri-strips as an adjunct to the stapled line in LVRS patients. In order to minimise individual patient bias we elected to allow patients to act as their own control.

## Conclusion

This pilot randomised self controlled trial demonstrates the comparable efficacy and safety of BioGlue in LVRS compared to Peri-strips reinforcement of staple lines. There is a trend favouring the BioGlue treated side in terms of reduction in air leak, chest drainage volumes and duration of chest drainage and significant absence of complications in the BioGlue treated sides. We need a large sample size to validate these results.

## Abbreviations

LVRS: Lung volume reduction surgery; HRCT: High Resolution CT scan; GIA: Gastrointestinal anastamosis; NETT: National Emphysema Treatment Trial; PTFE: Poly-tetrafluoroethylene.

## Competing interests

The study was supported by an educational grant from Cryolife Europa.

## Authors' contributions

SR was involved with study design, collected the data, performed the data analysis and authored the manuscript. BN was involved in study design and coauthored manuscript. PN collected data. ML collected data and co-authored manuscript. MSK performed data analysis and co authored manuscript. PBR is the principal investigator, devised the study and co authored the manuscript. All authors have read and approved the manuscript.
